# Sepsis-Induced Coagulopathy: An Update on Pathophysiology, Biomarkers, and Current Guidelines

**DOI:** 10.3390/life13020350

**Published:** 2023-01-28

**Authors:** Andreas G. Tsantes, Stavroula Parastatidou, Emmanuel A. Tsantes, Elli Bonova, Konstantina A. Tsante, Petros G. Mantzios, Aristeidis G. Vaiopoulos, Stavros Tsalas, Aikaterini Konstantinidi, Dimitra Houhoula, Nicoletta Iacovidou, Daniele Piovani, Georgios K. Nikolopoulos, Rozeta Sokou

**Affiliations:** 1 Laboratory of Haematology and Blood Bank Unit, “Attiko” Hospital, School of Medicine, National and Kapodistrian University of Athens, 12462 Athens, Greece; 2 Microbiology Department, “Saint Savvas” Oncology Hospital, 11522 Athens, Greece; 3 Neonatal Intensive Care Unit, 3rd Department of Paediatrics, “Attiko” Hospital, 12462 Athens, Greece; 4 Department of Biomedical Sciences, Humanitas University, Pieve Emanuele, 20090 Milan, Italy; 5 Neonatal Intensive Care Unit, “Agios Panteleimon” General Hospital of Nikea, 18454 Piraeus, Greece; 6 Neonatal Department, National and Kapodistrian University of Athens, Aretaeio Hospital, 11528 Athens, Greece; 7 IRCCS Humanitas Research Hospital, 20089 Rozzano, Italy; 8 Medical School, University of Cyprus, Nicosia 2029, Cyprus

**Keywords:** sepsis, coagulation, disseminated intravascular coagulation, sepsis-induced coagulopathy, laboratory evaluation, guidelines

## Abstract

Significant cross talk occurs between inflammation and coagulation. Thus, coagulopathy is common in sepsis, potentially aggravating the prognosis. Initially, septic patients tend to exhibit a prothrombotic state through extrinsic pathway activation, cytokine-induced coagulation amplification, anticoagulant pathways suppression, and fibrinolysis impairment. In late sepsis stages, with the establishment of disseminated intravascular coagulation (DIC), hypocoagulability ensues. Traditional laboratory findings of sepsis, including thrombocytopenia, increased prothrombin time (PT) and fibrin degradation products (FDPs), and decreased fibrinogen, only present late in the course of sepsis. A recently introduced definition of sepsis-induced coagulopathy (SIC) aims to identify patients at an earlier stage when changes to coagulation status are still reversible. Nonconventional assays, such as the measurement of anticoagulant proteins and nuclear material levels, and viscoelastic studies, have shown promising sensitivity and specificity in detecting patients at risk for DIC, allowing for timely therapeutic interventions. This review outlines current insights into the pathophysiological mechanisms and diagnostic options of SIC.

## 1. Introduction

**Figure 1:**
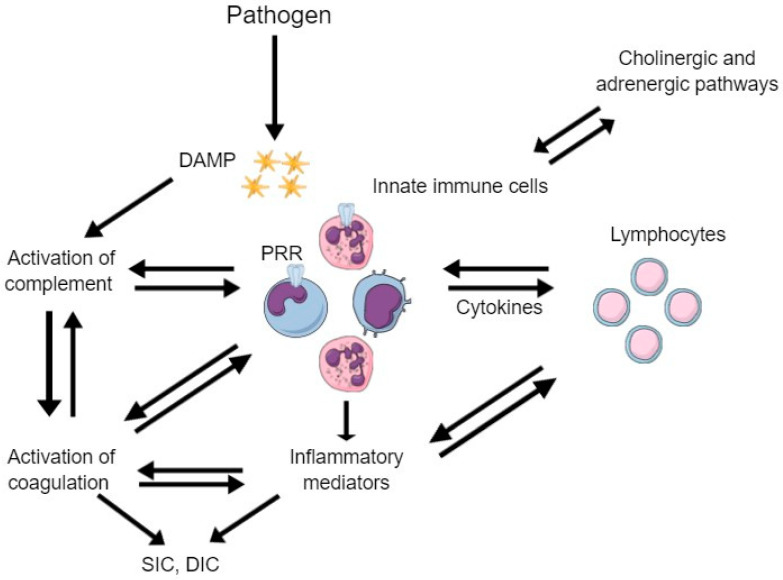
The main pathogenetic mechanisms involved in sepsis. Following pathogen invasion, innate immune cells recognize antigens through pattern-recognition receptors (PRRs). Activated innate immune cells release cytokines and inflammatory mediators to activate lymphocytes and the coagulation cascade, resulting in sepsis-induced coagulopathy and disseminated intravascular coagulopathy. Moreover, damage-associated molecular patterns (DAMPs) activate complement, which also results in activation of the coagulation cascade.

The traditional definition of sepsis is a systemic inflammatory response syndrome induced by infection. In 2016, this definition was updated by the Third International Consensus Definitions for Sepsis and Septic Shock to “*a life-threatening organ dysfunction caused by a host’s dysfunctional response to infection*” [1]. The current concept highlights the risk of severe morbidity and mortality due to the dysregulated host response rather than the infection itself. Organ dysfunction is defined as an increase in the sequential organ failure assessment (SOFA) score by at least 2 points. Our understanding of sepsis mechanisms and pathophysiology has greatly improved over the last few years, while contemporary and more efficient diagnostic tools are currently being developed (Figure 1). However, the pathobiology of sepsis has not been fully elucidated, and there is no gold standard diagnostic test. Sepsis is a complex clinical syndrome with a prevalence of approximately 2.5 per 1000 individuals in the Western world, and an annual increase of 8.7% over the last 20 years, partly due to the aging of the population [2]. Nineteen million cases of sepsis are diagnosed annually, with almost 5 million deaths, rendering it a leading cause of mortality globally [3].

**Figure 2:**
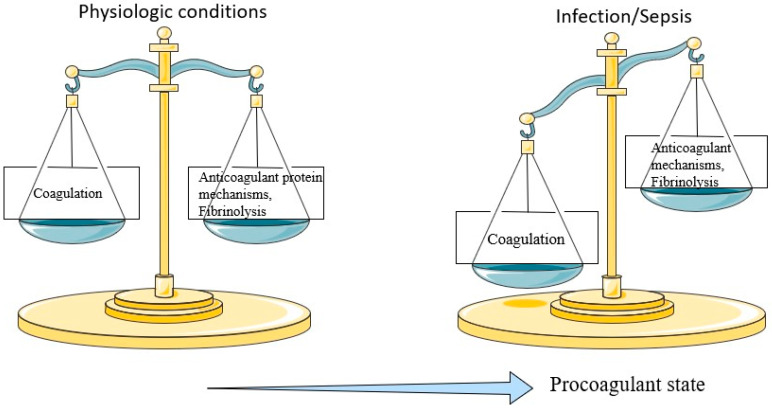
Derangement of the hemostatic balance in sepsis includes hyperactivation of the coagulation process and suppression of the anticoagulant mechanisms such as fibrinolysis and release of anticoagulant factors.

Infection, and sepsis, in particular, induce multiple and complex derangements in many systems, including the coagulation cascade. The vast majority of septic patients present with hemostatic abnormalities, ranging from subclinical coagulopathy to fulminant disseminated intravascular coagulation (DIC) [4]. During the initial stages of infection, coagulation operates as a natural defense mechanism, attempting to confine the responsible pathogen and prevent its spread into systematic circulation. However, in advanced and severe infections, as in sepsis, mass inflammatory cytokine production and release into the circulation lead to excessive activation of the coagulation process, fibrinolysis impairment, and suppression of anticoagulant mechanisms [5]. The hemostatic balance is significantly deranged in sepsis. The coagulation process is activated, while anticoagulant mechanisms, including fibrinolysis and anticoagulant factors, are suppressed (Figure 2). Consequently, septic patients are prone to a prothrombotic state through 4 main mechanisms: extrinsic pathway activation, cytokine-induced coagulation amplification, anticoagulant pathways suppression, and fibrinolysis impairment [6]. Laboratory assessment of hemostatic disorders in sepsis is based on conventional tests, including absolute platelet count, prothrombin time (PT), international normalized ratio (INR), fibrin degradation products (FDPs), such as d-dimers and fibrinogen levels, and nonconventional assays, including the measurement of anticoagulant proteins and nuclear material levels, and viscoelastic studies [2].

This review outlines current insights into the pathophysiological mechanisms and diagnostic options of sepsis-induced coagulopathy, focusing on potential biomarkers of interest and relevant existing guidelines. This study aims to review the current literature regarding the following key questions: (1) What are the main pathogenetic mechanisms involved in sepsis-induced coagulopathy? (2) What are the diagnostic criteria for sepsis-induced coagulopathy? (3) Are there any recent advances in the laboratory evaluation and treatment of sepsis-induced coagulopathy?

## 2. Hemostatic Abnormalities in Sepsis

The tissue factor pathway is the initial and main trigger for coagulation activation in sepsis. The tissue factor is located in the vascular endothelium, and its exposure to circulation in case of endothelial damage leads to activation of the extrinsic coagulation pathway [5]. However, this is not the primary activating mechanism in sepsis. Tissue factor is also present in inflammatory cells, mainly the monocytes and other circulating macrophages. In case of infection, these cells are activated through pattern-recognition receptors (PRRs) by pathogen-associated molecular patterns (PAMPs) and damage-associated molecular patterns (DAMPs) [7]. PAMPs are molecules that circulate following the destruction of pathogens or can be released by live pathogens, while DAMPs are host cellular components released when cells are lysed. PAMPs and DAMPs are recognized and bound to specific surface PRRs in monocytes and macrophages. Following PAMPs/DAMPs recognition by the PRRs on the surface of monocytes and macrophages, these cells are activated and release cytokines and chemokines, which subsequently activate neutrophils, platelets, and endothelial cells. Moreover, activated monocytes release extracellular vesicles that express procoagulant tissue factor and phosphatidylserine on their surfaces. Therefore, tissue factor is released into the circulation, and the extrinsic coagulation pathway is activated. Neutrophils also play a significant role in the activation of the coagulation cascade through the expression of the tissue factor and the release of chemical mediators and proteins. Another important mechanism through which neutrophils activate the coagulation cascade is the release of neutrophil extracellular traps (NETs). NETs are particles consisting of histones, procoagulant DNA, and other DAMP, released following the pathogen’s invasion to limit the infection. However, these NETs are highly prothrombotic, contributing to the procoagulant state of infection.

The activation of coagulation is further enhanced via the release of inflammatory cytokines and antigenic products [2]. Bacterial endotoxins, which are circulating bacterial cell wall fragments, are strong stimuli for the activation of coagulation cascade and DIC syndrome, particularly in Gram-negative infections. Endotoxins bind to monocyte and endothelial cells, leading to the release of coagulation-inducing cytokines and triggering tissue factor pathways [8]. Endotoxins, in this case, either bind to membrane-associated PRR or Toll-like receptors of these cells, or if they are present in cytosol, bind to and are detected by Caspase 11.

In the early stages of sepsis, coagulation activation is counteracted by three anticoagulant pathways: antithrombin, protein C, and tissue factor pathway inhibitor [2]. As sepsis progresses, these three mechanisms are deranged, resulting in a hypercoagulable state. Specifically, antithrombin, an important anticoagulant mechanism that inhibits thrombin and factor Xa, is decreased in sepsis because of reduced synthesis and increased degradation through proteases and neutrophil elastases [6]. Normal activation of protein C is mediated through the thrombin–thrombomodulin complex on endothelial cells leading to inhibition of factors Va and VIIIa. Additionally, the endothelial protein C receptor (EPCR) is expressed on the surface of endothelial cells and further enhances protein C activation [9]. However, protein C levels are suppressed in sepsis. Finally, the tissue factor pathway inhibitor (TFPI) inactivates the tissue factor–factor VIIa complex and is produced by several cells, including endothelial cells, but its levels are decreased in sepsis resulting in dysregulated inactivation of the tissue factor–factor VIIa complex.

In sepsis, fibrinolytic activity depends on the balance between the tissue plasminogen activator (t-PA) and tissue plasminogen activator inhibitor PAI-1 [10]. T-PA facilitates fibrinolysis through fibrin degradation by plasmin, while PAI-1 inhibits fibrinolysis. At the early transient phase of sepsis, fibrinolysis is increased because of the conversion of plasminogen to plasmin by the enhanced effect of t-PA. Subsequently, impairment of fibrinolysis ensues as PAI-1 is increased, thrombin-activatable fibrinolysis inhibitor (TAFI) levels are elevated, and so are plasma levels of nuclear products. Impairment of fibrinolysis contributes to the hypercoagulable state observed in sepsis [11]. Since coagulation serves as a defense mechanism, bacterial pathogens are confined in a fibrin network at the infection site, limiting the spread to adjacent tissues and systemic circulation. In this context, impairment of fibrinolysis is useful to some extent, but at the same time, it bears negative consequences [12]. Pathogens are prevented from disseminating to other tissues at the expense of unburdened oxygen flow to these tissues, which leads to hypoxia.

## 3. Inflammation–Hemostasis Cross Talk

**Figure 3:**
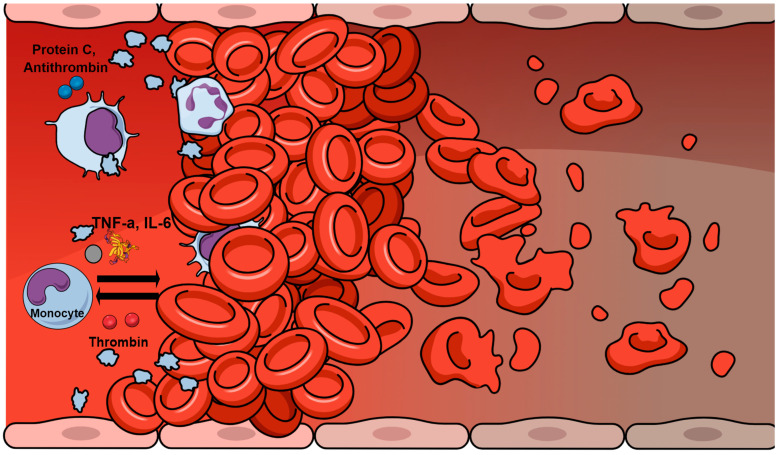
Main mechanisms involved in hemostasis–inflammation cross talk. Pro-inflammatory mediators such as tumor necrosis factor-a (TNF-a) and interleukin-6 (IL-6) are released by monocytes and macrophages to induce the coagulation cascade, while transmembrane receptors on monocytes can be activated by thrombin and other coagulation factors to trigger the release of pro-inflammatory cytokines. Moreover, antithrombin can directly bind to inflammatory cells and suppresses the expression of cytokine receptors, while also activated protein C downregulates endotoxin-induced production of cytokines by monocytes and macrophages.

In sepsis, a bi-directional interplay between inflammation and coagulation is developed, resulting in a self-sustaining cycle (Figure 3) [2]. High levels of pro-inflammatory mediators are present, most of which, including tumor necrosis factor-a (TNF-a) and interleukin-6 (IL-6), induce the coagulation cascade. At the same time, various hemostatic factors further sustain and augment the inflammatory process. Coagulation proteins interact with cell receptors, resulting in the modification of inflammatory pathways [5]. This regulatory action is largely mediated by protease-activated receptors (PARs) [13]. PARs are transmembrane receptors mainly activated by thrombin and other coagulation factors to trigger the release of pro-inflammatory cytokines [14]. Additionally, interactions occur between hemostasis and the complement, which plays an important role in DIC [15].

The significant cross talk also exists between anticoagulant factors and inflammatory mediators [5]. Antithrombin directly binds to inflammatory cells and suppresses the expression of cytokine receptors. Additionally, activated protein C has been shown to downregulate endotoxin-induced production of TNF-α, IL-1β, IL-6, and IL-8 by monocytes and macrophages. Inhibition of leukocyte activation may be another role of activated protein C [16].

## 4. Sepsis-Induced Coagulopathy and Disseminated Intravascular Coagulation

DIC is characterized by a dysfunctional systemic activation of the coagulation cascade leading to excess thrombotic and hemorrhagic complications resulting from intravascular fibrin formation, microangiopathic thrombosis, and subsequent depletion of coagulation factors and platelets [5]. Clinically evident hemostatic disorders occur in approximately 50% to 70% of septic patients, while 35% of these patients develop DIC [17]. Over three decades ago, the Scientific Subcommittee (SSC) of the International Society on Thrombosis and Haemostasis (ISTH) on DIC defined the condition as “*an acquired syndrome characterized by the intravascular activation of coagulation with loss of localization arising from different causes*” [18]. Although triggered by different underlying disorders, including sepsis and noninfectious causes such as trauma and malignancies, the common feature of DIC is the systemic activation of coagulation. This activation is induced by endothelial damage and may culminate in tissue vascular hypoperfusion and subsequent multiple organ failure [19]. Coagulopathy in septic patients progresses from an initially compensated derangement of the hemostatic system, termed non-overt DIC, to overt DIC, a totally decompensated coagulation state [20].

The first diagnostic criteria for DIC were introduced in 1983 by the Japanese Ministry of Health and Welfare. They included both clinical characteristics and laboratory variables such as platelet count, PT, FDPs, and fibrinogen [21]. Later, the ISTH DIC SSC recommended criteria for the diagnosis of overt DIC, focusing on laboratory parameters [18]. D-dimers were included in the criteria for the first time, while the significance of platelet count was reduced, and the importance of fibrin-related markers was increased. According to the ISTH DIC SSC criteria, elevated levels of FDPs or d-dimers, decreased platelet count, prolonged PT, and decreased fibrinogen levels are consistent with overt DIC (Table 1). Other DIC scoring systems have also been employed, including the Japanese Association for Acute Medicine (JAAM) DIC diagnostic criteria [22]. Platelet count, FDP concentration, PT, and systemic inflammatory response are among the JAAM DIC criteria.

Sepsis represents a condition where prompt diagnosis and initiation of treatment are of the utmost importance. The DIC score has shown a good predictive value for mortality; however, by the time of detection, patients are already at an advanced and irreversible stage of coagulopathy, beyond the optimal timeframe for therapeutic intervention [23]. Early identification of patients with sepsis-associated coagulopathy prior to progressing to this phase of hemostatic derangement would be ideal for the initiation of anticoagulant treatment [24]. Thus, the ISTH DIC subcommittee suggested simple diagnostic criteria for sepsis-induced coagulopathy (SIC) composed of only three parameters: platelet count, PT or INR, and SOFA score [25]. The presence of sepsis is confirmed by the inclusion of the SOFA score, reflecting the updated sepsis definition. DIC associated with sepsis is characterized by impairment of fibrinolysis, resulting from excessive production of PAI-1 [26,27]. This potentially leads to a prothrombotic state and organ dysfunction due to tissue hypoperfusion [28,29]. In contrast, in non-sepsis DIC, suppression of fibrinolysis is rare and systemic bleeding often occurs [30]. Therefore, reduced fibrinogen levels are not a common or specific finding in sepsis, in contrast to thrombocytopenia and PT prolongation. Finally, FDPs and d-dimers were not included in the SIC score as they lacked correlation with the severity of sepsis [31].

Comparing the ISTH overt DIC and SIC scoring systems, it became evident that the SIC score was twice as sensitive as the overt DIC score and that SIC always preceded overt DIC [32]. The SIC score was also validated against the JAAM DIC criteria and demonstrated similar prognostic value [33].

Diagnosis of either SIC or overt DIC may be useful in identifying patients who would benefit from therapeutic anticoagulant intervention [34]. A “two-step” sequential scoring system was developed by the ISTH for the identification of patients with sepsis-associated coagulopathy (Table 1). Patients are initially screened with the SIC score, and if the criteria for SIC are met, the overt DIC score is then calculated. This approach could increase the possibility of timely identification of suitable candidates for anticoagulant treatment [21].

## 5. Conventional Hemostatic Tests and Markers in Sepsis

In case of sepsis, well-established findings emerge on conventional coagulation assays. These findings include thrombocytopenia, prolongation of PT, increased levels of FDPs, and reduced fibrinogen levels. Such abnormalities often present in the setting of DIC and are not usually observed before its development, during the initial stages of sepsis.

### 5.1. Thrombocytopenia

Sepsis is typically associated with thrombocytopenia, despite the release of pro-inflammatory mediators and thrombopoietin, both of which induce platelet production [35,36]. The vast majority of sepsis patients develop thrombocytopenia, an established independent predictor of poor outcomes in sepsis [37,38]. In the early phase of sepsis, platelets, following their activation, aggregate with leucocytes to form platelet–leucocyte aggregates, leading to increased sequestration in the spleen [39]. Moreover, infections can stimulate platelet activation and aggregation and cause thrombocytopenia, either directly or through cell destruction, inflammation, and clot formation [40]. The phagocytosis of thrombocyte progenitors by monocytes due to macrophage colony-stimulating factor (M-CSF) further contributes to the low platelet count. In addition, altered production of platelets is observed in sepsis with a significant release of precursor cells, and the immature platelet fraction (IPF) correlates well with sepsis severity scores [41].

### 5.2. Fibrin Degradation Products

Assessment of fibrin-related biomarkers includes tests detecting FDPs, such as d-dimers, and assays detecting precursor fibrin formation products at an intermediate stage between fibrinogen and final stable fibrin, such as soluble fibrin monomers.

FDPs, such as protein fragments X, Y, and D, mainly result from the degradation of three components in the fibrinogen–fibrin cycle: the initial fibrinogen, the soluble precursor fibrin, and the cross-linked fibrin—the final stable form of fibrin. FDPs have been found in up to 99% of sepsis patients. However, these tests lack specificity, as they cannot discriminate degradation products deriving from final cross-linked fibrin, intermediate soluble fibrin, or initial fibrinogen [42]. FDP levels are measured by immunoenzyme assay ELISA or latex agglutination methods, allowing for point of care evaluation.

D-dimers are also FDPs, but they only result from proteolysis of the final cross-linked fibrin, and their levels are not affected by fibrinogen degradation products [5]. Consequently, d-dimers are the most specific marker among FDPs for assessing DIC. Detection of d-dimers indicates that thrombin induces the conversion of fibrinogen to fibrin monomer and that fibrin is cross-linked by activated factor XIII and subsequently degraded by plasmin [43]. Although d-dimers are FDPs and are associated with fibrinolysis, high levels reflect concomitant activation of coagulation [44]. Therefore, d-dimers may indicate increased formation rather than degradation of fibrin. In fact, increased d-dimers levels are observed in thromboses and are also included in DIC scores where severe sepsis is correlated with hypofibrinolysis. Thus, in sepsis, d-dimers are elevated because of both the activation of the coagulation cascade and the hyperfibrinolysis observed in the early phase of infection. However, in advanced sepsis, d-dimers may be normal. This is attributed to a significant inhibition of fibrinolysis, which prevents the formation of d-dimers despite increased fibrin formation. There is evidence that the use of d-dimers for the diagnosis of DIC and risk stratification in severe sepsis may be misleading, as normal levels of d-dimers were associated with higher mortality rates [43,44].

On the other hand, soluble fibrin monomers could be effective markers of intravascular fibrin formation in DIC [45]. Plasma levels of soluble fibrin monomers reflect intravascular fibrin formation and are not affected by extravascular fibrin formation that may accompany local inflammation or trauma. Available clinical trials demonstrate that soluble fibrin monomers exceeding a threshold level allow for DIC accurate diagnosis. To date, no reliable test is available for the quantification of soluble fibrin levels.

### 5.3. Fibrinogen

In general, low serum fibrinogen levels are considered a primary index of coagulation disorders in sepsis [46]. However, fibrinogen is an acute-phase reactant, and its levels are increased because of inflammation. As a result, fibrinogen levels may remain within the normal reference range for a long period of time after its continuing consumption. Sequential fibrinogen measurements might be more helpful, with higher diagnostic accuracy [47].

### 5.4. Standard Coagulation Tests

Standard coagulation tests, including PT and activated partial thromboplastin time (aPTT), have traditionally been used to assess the hemostatic system [48]. Nevertheless, they fail to fully reflect the hemostatic derangement incited by sepsis [49]. Abnormal values of standard coagulation tests are only observed late in the course of sepsis when the hypocoagulable hemostatic status emerges and is detected by these tests. Additionally, they assess part of the hemostatic mechanism, as they are based on plasmatic components of the coagulation system, while the crucial contribution of cellular components is not evaluated. Standard coagulation tests primarily assess the activity of procoagulant factors without taking into account the simultaneous activation of the anticoagulant mechanism.

## 6. Nonconventional Hemostatic Tests and Markers in Sepsis

Nonconventional tests have been applied in the diagnosis of coagulation derangement associated with sepsis.

### 6.1. Viscoelastic Tests

Thromboelastography (TEG) and rotational thromboelastometry (ROTEM) are viscoelastic, whole-blood, point of care tests that provide a comprehensive evaluation of the hemostatic process from coagulation initiation and clot formation to clot dissolution and fibrinolysis [50,51]. The contribution of both plasmatic and cellular components of coagulation are assessed through these methods. Viscoelastic tests may be useful for completing the gaps of the standard coagulation tests in evaluating the hemostatic status of septic patients [49]. Thus, numerous studies have focused on the use of viscoelastic tests for the detection of the hemostatic changes accompanying sepsis. Studies in subpopulations, including pediatric and neonatal septic patients, have also been conducted [52,53,54]. The prevalence of septic patients with hemostatic disorders detected by TEG/ ROTEM ranged between 43% and 100% [55]. There is heterogeneity in the design, conduction, and results of the studies [56,57]. Both hypocoagulability and hypercoagulability were reported, reflecting the pathophysiology of coagulopathy in sepsis and DIC. As a dynamic process, coagulopathy associated with sepsis evolves rapidly, and the timing of the testing greatly affects TEG/ ROTEM results. Sequential measurements may more accurately depict the progress of SIC, from hypercoagulability in the early stages to subsequent development of DIC and hypocoagulability. Viscoelastic tests can also detect impairment in fibrinolysis, as reported in several studies in sepsis patients [55,58].

Viscoelastic assays can detect the early activation of coagulation that leads to hypercoagulability when standard coagulation test results are still within normal limits [55]. ROTEM has been used to distinguish between septic patients with normal coagulability, hypercoagulability, and hypocoagulability. Both hypocoagulable and hypercoagulable hemostatic profile in ROTEM was correlated with significantly higher mortality risk [59]. In another study, patients with overt DIC presented a hypocoagulable profile in ROTEM, while septic patients without overt DIC tended to demonstrate hypercoagulability [60]. All of these patients had abnormal conventional coagulation tests, and the use of ROTEM could help prevent procoagulant interventions in patients with a hypercoagulable status not detected by standard coagulation assays. One hundred patients with sepsis, severe sepsis, or septic shock were assessed by ROTEM assay [61]. Maximum clot firmness (MCF) was higher in the sepsis and severe sepsis groups compared with the healthy control group. Normal MCF and prolonged clot development were observed in case of septic shock. These results indicate hypercoagulability in patients with sepsis or severe sepsis and hypocoagulability in the more advanced phase of septic shock. Interestingly, fibrinolytic activity was positively correlated with 28-day mortality. TEG parameters have been used for the development of a score to predict DIC [62]. Furthermore, ROTEM clotting time (CT) variable correlated strongly with the JAAM DIC score and performed well in predicting DIC [63].

A recent meta-analysis concluded that viscoelastic assays are reliable and useful for predicting DIC associated with sepsis and assessing mortality risk in severe sepsis [56]. It seems that both the hypocoagulable profile and transition towards hypocoagulability could identify patients at risk for developing DIC. Serial and combined viscoelastic and conventional coagulation testing could optimize DIC detection and management and improve survival and outcome.

### 6.2. Measurement of Nuclear Materials

Sepsis causes cellular activation and damage and the subsequent release of nuclear materials into circulation [15]. Nuclear materials include high mobility group box protein-1 (HMGB1), nucleosomes, histones, cell-free DNA, and neutrophil extracellular traps (NETs). Nuclear materials can directly trigger the expression of tissue factor in macrophages and endothelial cells. Tissue factor expression and subsequent activation of the extrinsic coagulation cataract is mediated by the activation of TLR2/4 receptors and the nuclear factor-kappa B (NF-κB) and activator protein 1 (AP-1) pathways [64].

HMGB1 serves as a marker of cell damage, inflammation, and thrombosis [65]. Acting on surrounding cells through receptors, it mediates the migration of leucocytes and the production of pro-inflammatory cytokines [66]. HMGB1 also promotes tissue factor expression on the surface of monocytes and reduces the anticoagulant activity of thrombomodulin, facilitating microvascular thrombosis through these pathways. High HMGB1 levels have been observed in patients with sepsis and DIC and are associated with poor prognosis and increased mortality [67].

Nucleosomes are complexes formed of 147 base pairs of DNA wrapped around a protein core of histones [68]. Nucleosomes are released into circulation with apoptotic cell death [69]. Elevated nucleosome levels were reported in various conditions, including malignancies, cerebral stroke, sepsis, and septic shock [70]. Increased plasma histone levels are observed in patients with sepsis and DIC and correlate positively with mortality [71]. In a preclinical model, extracellular histones triggered platelet aggregation, leading to consumptive coagulopathy, thrombosis, and bleeding. The prothrombotic action of histones is inhibited by recombinant thrombomodulin, which has been used therapeutically in DIC. Apart from being a biomarker of endothelial damage and activation of hemostasis, histone levels are associated with SOFA scores [72].

Plasma cell-free DNA demonstrated high prognostic value in patients with severe sepsis, superior to the multiple organ dysfunction (MODS) and the acute physiology and chronic health evaluation (APACHE) II score [73].

NETs are extracellular components of neutrophils, formed by DNA, histones, and granule proteins, and exhibit significant prothrombotic and pro-inflammatory properties [74].

### 6.3. Measurement of Anticoagulant Proteins

Antithrombin is one of the most important physiological anticoagulants and inhibits the intrinsic (factor XIa), extrinsic (factor VIIa), and common coagulation pathways (factor Xa, thrombin). In sepsis and DIC, antithrombin levels are decreased because of reduced synthesis, increased degradation by neutrophil elastase, and excess consumption resulting from the overproduction of thrombin [75]. Reduction in antithrombin levels is associated with disease severity in sepsis-induced DIC [76]. A decrease in antithrombin activity below 80% was reported in septic patients without multiple organ dysfunction [77]. This level reaches 60% in patients with multiple organ dysfunction, while progress to DIC further reduces antithrombin activity to approximately 40%. Increased mortality was demonstrated with low antithrombin levels in adult and pediatric septic patients [78,79].

Another anticoagulant protein, protein C, is naturally activated to inhibit thrombin formation through both extrinsic and intrinsic pathways [6]. Sepsis is characterized by the depletion of protein C and impaired production of activated protein C, rendering their levels potential prognostic biomarkers in this setting [80]. Activated protein C binds to its endothelial receptor (EPCR), which is released into the circulation following cell damage. This soluble EPCR was also studied as an index of disease progression and outcome in septic patients [81].

Thrombomodulin is an anticoagulant protein produced by endothelial cells and binds to thrombin, converting protein C into its activated form [82]. Thrombomodulin has a key anti-inflammatory, anticoagulant, and antifibrinolytic role. It inhibits leucocyte adhesion to endothelial cells, prevents complement activation, modifies cytokine generation, and degrades HMGB1 [83]. Thrombomodulin is cleaved by neutrophil elastase and circulates in its soluble form [84]. High plasma thrombomodulin levels are correlated with increased mortality in patients with sepsis and DIC [85]. Elevation of soluble plasma thrombomodulin with a concomitant decrease in endothelial surface thrombomodulin is observed in sepsis-induced DIC [86].

The natural anticoagulant TFPI inhibits the activation of tissue factor and the extrinsic coagulation pathway. Depletion of TFPI is associated with increased susceptibility to the development of DIC [6]. TFPI binds to endothelial heparin-like molecules, and its circulating levels are lower compared with antithrombin and activated protein C. Moreover, a significant part of TFPI is inactivated by binding to the lipoprotein. Consequently, TFPI has not emerged as a sensitive marker of DIC.

### 6.4. Other Markers of Interest

The thrombin–antithrombin (TAT) complex is a marker of thrombin generation, which could be used to identify patients with DIC, as excess thrombin formation is a key feature of this condition [46]. Higher TAT levels were reported in patients with DIC on admission or in those who developed DIC compared with patients without DIC [87]. Raised TAT levels were correlated with increased mortality in sepsis patients [26]. A combination of TAT with other markers has a higher prognostic value than each molecule alone.

Although fibrinolysis is impaired in sepsis-induced DIC, this is not detected by current coagulation parameters. The antifibrinolytic protein PAI-1 has been extensively investigated as a potential marker of hypofibrinolysis in septic patients with DIC [46]. PAH-1 levels can identify patients with DIC and predict DIC development [88,89]. Similar to TAT, combining PAI-1 with other biomarkers increased the performance ability of PAI-1 [26]. High PAI-1 was associated with increased mortality risk [90].

Endogenous thrombin potential—an assay indicative of thrombin generation—was evaluated in sepsis. Results were conflicting, and further research is warranted. However, recent evidence suggested a correlation between increased infection severity and decreased ability of thrombin generation [91]. This biomarker could possibly serve in the prediction of multiorgan dysfunction development and poor outcome in septic patients [92].

Prothrombin fragment 1.2 (F1.2) is an activation peptide generated when prothrombin is converted to thrombin [93]. Its use as a marker of thrombin generation was published and included in the DIC criteria of the Japanese Society on Thrombosis and Hemostasis (JSTH) [94].

ADAMTS-13 is a von Willebrand factor-cleaving protease regulating the size of von Willebrand factor multimers [95]. Cleaving von Willebrand multimers decrease their prothrombotic properties, as larger molecules have enhanced hemostatic competency [96]. ADAMTS-13 deficiency, leading to ultra large von Willebrand factor multimers and thrombotic microangiopathy in sepsis, was associated with sepsis severity and poor prognosis.

Fourteen conventional and nonconventional biomarkers, including platelet count, PT, aPTT, fibrinogen, FDPs, TAT, protein C, plasminogen, and PAI-1, were evaluated for early diagnosis of DIC in a study by Koyama et al. [26]. TAT, PAI-1, and protein C on admission discriminated well between patients with and without overt DIC, while TAT and PAI-1 were also significant predictors of 28-day mortality. Apart from predicting prognosis, certain markers can additionally be used to monitor treatment in patients with DIC [15]. Furthermore, some of them, including antithrombin and recombinant soluble thrombomodulin, were also studied and used for the treatment of DIC.

## 7. From Guidelines to Clinical Practice

Currently, there is no established therapy for sepsis-induced coagulopathy. International Surviving Sepsis Campaign guidelines on the management of sepsis and septic shock have recently been published [97]. On the basis of these guidelines, pharmacologic thromboprophylaxis with low molecular weight heparin is strongly recommended for patients with sepsis or septic shock. Moreover, in the latest Japanese Surviving Sepsis Campaign guidelines of 2020, the early detection of DIC is encouraged, and the use of anticoagulants for sepsis-associated DIC is weakly recommended [98].

Several treatment strategies for SIC have been evaluated, mainly directed at suppressing the prothrombotic effects, such as the administration of heparin/heparinoids or anticoagulant proteins (Table 2) [99,100]. The value of recombinant thrombomodulin in sepsis-induced coagulopathy was recently evaluated in a multinational, randomized controlled phase III trial (including patients with a platelet count < 50 × 10^9^/L and a prothrombin time ratio > 1.4). The authors of this study reported that the 28-day mortality improved by 2.6% in 800 septic patients, although this difference in the mortality rate was not statistically significant. [101]. During the last few years, several anticoagulant agents, including antithrombin, have been tested in an attempt to reduce mortality in patients with sepsis; however, they have largely failed to prove effective [101]. It should be noted, though, that all these trials targeted septic patients and not patients with SIC. Antithrombin is not recommended by the international sepsis guidelines. In contrast, post hoc analysis of databases has indicated the beneficial effects of anticoagulants in certain subgroups of septic patients with DIC [102]. A possible explanation for this discrepancy lies in the heterogeneity of patients and the timing of anticoagulant treatment in large-scale trials. Precision medicine principles should be applied to select suitable candidates for anticoagulation intervention among sepsis patients with SIC.

## 8. Conclusions

This updated review consolidates all the current knowledge regarding the pathogenesis of SIC while it includes all the recent updates regarding the diagnostic criteria for SIC based on international scientific societies. Moreover, this comprehensive review includes an extensive discussion of all conventional and nonconventional biomarkers for SIC. Sepsis-induced coagulopathy often complicates the course of patients, with potentially adverse outcomes and increased mortality. Hypercoagulability is a well-established pathophysiological feature of sepsis-induced coagulopathy, with a shift towards hypocoagulability only in the late sepsis stages, after the development of DIC. Traditional coagulation tests can detect late-stage hypocoagulability but are not sensitive to identify early-phase hypocoagulability. A gap exists in the diagnosis of hemostatic derangement in sepsis. The definition of SIC was introduced to help identify septic patients before the establishment of DIC, during the early stages, which could be reversed with therapeutic interventions. In this context, investigating novel, specialized assays, such as thromboelastometry, is essential for patients with severe sepsis, as it may allow for early intervention during the hypercoagulability phase. Further research for diagnostic biomarkers and potential combinations to increase diagnostic accuracy is warranted.

## Figures and Tables

**Table 1:** International Society on Thrombosis and Haemostasis criteria for DIC and SIC diagnosis.

Item	Score	Overt DIC	SIC
		Range	Range
Platelet count (×10^9^/L)	2 1	<50 ≥50, <100	<100 ≥100, <150
FDP/D-dimers	3 2	Strong increase Moderate increase	- -
Prothrombin time (PT ratio)	2 1	≥6 s ≥3 s, <6 s	(>1.4) (>1.2, ≤1.4)
Fibrinogen (g/mL)	1	<100	-
SOFA score	2 1	- -	≥2 1
Total score for DIC or SIC		≥5	≥4

Abbreviations: DIC—disseminated intravascular coagulation; SIC—sepsis-induced coagulopathy; FDP—fibrin degradation products; SOFA—Sequential Organ Failure Assessment.

**Table 2:** Treatment options for sepsis-induced coagulopathy.

Treatment Options	Comments	References
Heparin and heparinoids	Their effectiveness in sepsis-induced coagulopathy is debatable and limited to preventing deep vein thrombosis.	Iba et al. [99]
Antithrombin, activated protein C, and tissue factor pathway inhibitors	Potential survival benefit with antithrombin administration.	Kienast et al. [100]
Fresh frozen plasma	No evidence supporting its use unless there are specific indications for bleeding or factor depletion beyond antithrombin	Iba et al. [99]
Recombinant thrombomodulin	It may improve overall mortality in patients with SIC	Vincent et al. [101]

## Data Availability

Not applicable.
